# The Changing Landscape of Arctic Traditional Food

**DOI:** 10.1289/ehp.118-a386

**Published:** 2010-09

**Authors:** Tim Lougheed

**Affiliations:** **Tim Lougheed** has worked as a freelance writer in Ottawa, Canada, since 1991. A past president of the Canadian Science Writers’ Association, he covers a broad range of topics in science, technology, medicine, and education

The earliest European explorers seeking a northwest passage to Asia did not know what to make of the indigenous inhabitants they encountered in what is now Canada. In the 1500s, Martin Frobisher thought they were Asians and took a number as slaves; none survived more than a few weeks in captivity. Later adventurers acquired a profound respect for the knowledge that had enabled Inuit (“the people”) to thrive for centuries in a harsh environment that left so many newcomers starving and frozen. When Norwegian Roald Amundsen reached the South Pole without incident in 1911, he credited his success to what he had learned from Inuit while carrying out a two-year magnetic survey of the eastern Arctic a decade earlier.

Above all, he learned how to eat under extreme polar conditions. While Robert Scott tackled the South Pole at exactly the same time, his expedition was doomed by sustenance on biscuits and tinned meat packed in lead-soldered cans. In contrast, the Norwegians followed Inuit practice to dine on the fatty flesh of freshly caught seal and penguin, obtaining key nutrients that prevented the onset of scurvy.^1^

Such a diet makes perfect sense in places that cannot furnish fruits and vegetables. Some Inuit in Canada’s high Arctic, as well as further south, continue to thrive in this way. But the way of life that served Amundsen so well has undergone a dramatic transformation during the ensuing century. North America’s “attic” still qualifies as remote, but transportation and communications technology have eliminated its isolation.

## Rapid Change from Old Ways

Today one-third of the world’s Inuit live in Canada, scattered in communities that range in size from a few dozen to several thousand. The Internet and satellite television have given these northern inhabitants an unprecedented awareness of the wider world, while a money-based economy has given them goods manufactured by that world. Snowmobiles and powerboats have largely replaced dogsleds and kayaks, just as high-powered rifles long ago replaced hunting spears. And where people once wrested their own food from the surrounding land or sea, today many buy groceries in a store, including processed and packaged products that would have been unknown a few decades ago.

Drastic and often disastrous shifts in diet can be traced to the Canadian government’s forced settlement of these formerly nomadic peoples in the years following World War II. The social upheaval generated within the resettled populations has been among the major challenges facing the province of Nunavut, which was created from the eastern portion of Canada’s Northwest Territories in 1999 as a dedicated Inuit territory.

“Generations [of Inuit] are now experiencing the consequences of a poor diet,” Geraldine Osborne, minister of Nunavut’s Department of Health and Social Services, wrote in the introduction to the government’s 2007 strategy for setting higher nutritional standards among Nunavummiut (people of Nunavut).^2^ “We cannot turn the clock back,” she acknowledged, “but we can optimize the resources now available, using both traditional and nutritious store food so that the basic dietary needs of all Nunavummiut are met.”

According to the government strategy, those efforts are to be grounded in a thorough knowledge of Inuit food and diet. Little of that knowledge has changed since it was passed along to Amundsen, but the scientific community is just beginning to analyze the many remarkable aspects of northern nutrition.

It has been some 25 years since Éric Dewailly learned how remarkable this subject could be. Now a community health researcher at Laval University in Quebec City, in the mid-1980s he was engaged in what was expected to be a straightforward survey of persistent organic pollutants (POPs) found in breast milk samples from the province of Quebec.^3^ For purely logistical reasons Dewailly and his colleagues planned to take samples from regional hospitals located in major population centers, ignoring outlying centers that would be harder to reach. Nor did that omission seem problematic, he recalls; no one at the time expected there to be any evidence of POPs in breast milk from these locations, which were thought to be unpolluted.

When the efforts of a local midwife made it possible to collect samples from smaller communities around Hudson Bay, the researchers accepted them, expecting these would serve as a kind of POP-neutral control group. Much to Dewailly’s surprise, however, these samples were seven times more contaminated than the others.^3^ Laboratory staff analyzed their own equipment for signs of cross-contamination from the other samples, until they were finally forced to conclude that even far-flung residents of the north were being exposed to these agents.

“This [illustrates] long-range transport of chemicals around the planet,” explains Dewailly. “The reason why Inuit were and are more exposed is just because of their dietary habits.”

The discovery cast those habits in a new light. Inuit and others were regularly subsisting on animals at the top of the food chain, animals that are also relatively long-lived. The prized fatty tissues of species such as seal can therefore concentrate POPs to a much greater degree than domestic livestock consumed elsewhere in the world. The implications are disturbing, given how perfectly the traditional diet is suited to life in the far north. As one Inuit elder put it, “When one eats seal, you are full all day. When you eat packaged foods, two hours later you get cold. If [you] eat Inuit food, you stay warm.”^4^

In order to assess the specific risks this situation posed to aboriginal populations, Canada’s federal government established its Northern Contaminants Program in 1991. This initiative funded research on the levels of metals, POPs, and other agents found in people and animals in the Arctic. A converted icebreaker traveled along Canada’s Arctic coast, at each stop becoming a temporary public health clinic. By 1992 there had been a major public health survey conducted in Nunavik, the official name for the area of Quebec north of the 55th parallel, whose population of around 11,000 is mostly Inuit. Dewailly went on to become a principal investigator for a second survey of the region in 2004, which captured changes in lifestyle, diet, and risk.^5^

In 2007 and 2008 support from an International Polar Year project made it possible to conduct a more focused survey, again traveling in a converted icebreaker to collect detailed information about some 2,500 children and adults living in 36 different communities. The findings, which are just beginning to be published in peer-reviewed journals, have established a firm foundation for taking stock of how well aboriginal populations have been coping with dramatic changes to their way of life. And while the results show the negative effects of a diet that has expanded to include sugar and saturated fats, the positive effects of the more traditional diet are also being demonstrated and emphasized.

## Health Survey Findings

Dewailly points out there has been some good news to report. Type 2 diabetes, which ravages some aboriginal populations in the rest of Canada, has not spiked in the same way amongst Inuit. Just 5% of participants in the 2007–2008 survey said they had been diagnosed as diabetic, compared with a national prevalence of 5.8%.^6^ The reported incidence of heart disease was similarly low, a longstanding observation that has been linked to the regular consumption of fish and sea mammals.^7^

The 2004 health survey revealed significant decreases in the amount of mercury, lead, and cadmium in participants’ blood. The change was most pronounced in lead levels, dropping 55% between the 1992 and 2004 surveys.^8^ Dewailly directly attributes this drop to the banning of lead shot ammunition for the hunting of most migratory game birds, which was implemented across Canada in 1999. A study published in 2003, which found high lead levels in umbilical cord blood of 475 Inuit infants born prior to the ban, also noted such pellets were commonly observed in the digestive system of Inuit during abdominal X-ray examinations, although food diaries of women revealed that their lead intake was well below established limits of tolerance.^9^ Indeed, a source identification study using stable lead isotope ratios confirmed that the use of lead shot in the harvesting of wild game was a major source of lead exposure for subsistence harvesting groups in Canada.^10^

Decreasing blood levels of mercury and cadmium—32% and 22% respectively—were not as clear-cut. The researchers regard the former as less of an environmental change than a dietary one. In other words, local wildlife may contain as much mercury as ever, but people are simply consuming fewer of these animals and taking in less of the metal. The authors did not regard the drop in cadmium as especially significant, instead linking the persistence of this metal to exceptionally high levels of smoking in the north.^8^

That conclusion was echoed by the 2007–2008 shipbound survey, which in one typical region found 82% of households had smokers, with an average of two per home. In that same survey, 29% of participants had parents diagnosed with cancer, and 16% had siblings diagnosed with cancer.^6^

With specific reference to cancer among Inuit, a survey of the 1989–2003 period identified dramatically different rates for various types of the disease, with equally significant distinctions amongst populations in different regions. The results showed generally high risks for lung, nasopharynx, and salivary gland cancer in comparison with non-Inuit groups.^11^

Meanwhile, incidence of breast and prostate cancer, which are among the most common around the world, remain very low among the Inuit—in a study of autopsied Inuit men in Greenland, Dewailly and his colleagues found almost no indication of prostate cancer, a finding they suggest could be related to the intake of wild food rich in omega-3 polyunsaturated fatty acids and selenium.^12^

The protective effects of these components could be expected to diminish as such food becomes a less prominent portion of diet. What that proportion might be was indicated in a 2006 federally administered survey of about 6,300 Inuit children and adults across the Arctic.^13^ The responses showed that in some 65% of homes, “country food” such as seal, caribou, whale, duck, fish, and berries accounted for at least half the food consumed in the household. That observation carried significant implications for leaders within the community.

“I am pleased to note that Inuit continue to harvest country food at a high rate,” says Mary Simon, president of Inuit Tapiriit Kanatami, a 29-year-old organization representing Inuit interests across Canada. “This, for Inuit, supports our culture and provides the healthiest food.”

## Fundamental Shifts

Nevertheless, other observers have cited the waning contribution of this food to over-all caloric intake. Researchers with McGill University and the University of Montreal set the role of traditional foods in this context. “Before colonial contact in the Americas, Indigenous Peoples had 100% of their dietary energy from their [traditional food] resources,” they wrote in 2004.^14^ “This pattern persisted in the Canadian Arctic until the advent of Hudson’s Bay stores at the turn of the twentieth century. Today only 10–36% of adult dietary energy is derived from [traditional foods].”

The rest of that dietary energy, they noted, comes from purchased foods that often have a much lower nutritional value. This includes items such as soft drinks, snack foods, and preprocessed meats in the form of ground beef, bacon, and frankfurters. In a further analysis published later in the same journal, these foods were found to dominate indigenous children’s diets.^15^ In that study, traditional foods accounted for less than 10% of food energy, while some 21% came from market foods identified as “fat” and another 20% from foods identified as “sweet.”

The results can be seen in the mouths of aboriginal children, as dentist Leonard Tsuji can attest. He spent the early part of his career filling far more than the average number of cavities and extracting thousands of teeth in Moose Factory, a community on James Bay that was originally established as a fur trading post in the 1600s. The aboriginal peoples in this area are Cree, rather than Inuit, but their lives in the roadless reaches of northern Ontario pose similar challenges with regard to food.

Now a professor in the Department of Environment & Resource Studies at the University of Waterloo, Tsuji and his colleague Rhona Hanning have spent the past decade collecting detailed dietary information on approximately 500 Cree schoolchildren. As overweight and obesity were found to be a problem, Tsuji and Hanning have initiated and augmented school-based nutritional programs for children in the western James Bay region. He can confirm the observation that most of the children rely on cheap calories from junk food, but he points out this is a rational move by people with few options.

“You’re preaching to them that they should be partaking in a more nutritious diet when it’s economically not feasible and it’s not available,” he says. Even if fruits and vegetables are available in the local store, the quality of these items is often unacceptable (e.g., they may be rotten), Tsuji says. In addition, their prices can be significantly higher than they would be in communities further south, while average income levels in the north are far lower.^16^

Traditional foods might appear to be an inexpensive or even “free” alternative, but that is not necessarily the case. “It’s expensive to get your guns, your boats, your snowmobiles,” Tsuji maintains. In fact, a snowmobile might cost US$7,000–10,000, a boat and motor US$10,000–15,000, and gasoline to run them could be upwards of US$5 a gallon. Even where these resources exist, he adds, they fundamentally alter the nature of food gathering.

“It’s not just the monetary value of the traditional foods, it’s the sociocultural aspects,” he says. “In the old days [hunters] would go out with their families for a month for the spring harvest of waterfowl. Many don’t do that anymore.”

Instead, mechanization has allowed Cree hunters to make quick trips out on the land, returning with harvests that they share with other community members. Nevertheless, waterfowl harvests have been erratic of late, as some hunters have suggested climate change has affected the timing of the spring migration of waterfowl as well as the migratory routes taken by the waterfowl, because of the longer presence of open water.

Climate change also appears to be one of the factors explaining the appearance of furunculosis—a disease caused by the temperature-sensitive bacterium *Aeromonas salmonicida*—in fish consumed for subsistence by the eastern James Bay Cree of northern Quebec. A temporal pattern was found to exist between the first appearance of furunculosis and regional temperature change in the region.^17^ Warming weather also causes once-frozen soil to shift unexpectedly, while the ice covering the ocean may not always be thick enough to support the weight of vehicles.

According to James Ford, a geographer at McGill University, it all adds to the risk faced by anyone venturing out to look for food—risks that many individuals no longer care to assume. “We’re seeing younger generations not developing the same skills as they once would, skills for safe and successful hunting,” he says. “In many cases we’re seeing young people who aren’t going hunting because it’s too dangerous. They don’t have the knowledge, they don’t have the skills, they don’t have the equipment.”

## Regaining Security

For some of these people, the significance of traditional foods may be fading. Marie-Pierre Lardeau, a scientist working in Ford’s group, has been studying the growing importance of food banks and soup kitchens found in the Arctic’s larger population centers. She concludes that climate change may mean little to those who depend on these services. More ominously, though, the very existence of these services raises questions about food security.

In an era when “security” has acquired a new gravitas, the term is no less politically loaded for representatives of the Inuit community. Their ancestors managed to feed themselves from the land and sea where Europeans perished. Yet a recent survey showed that nearly 70% of Inuit preschoolers surveyed lived in a household deemed food insecure.^18^ In this survey, primary caregivers reported a range of scenarios from worrying about running out of money to buy food to preschoolers not eating for a whole day because there was nothing to eat.

Such insights have prompted formal calls for programs to improve the pricing and availability of both market food and traditional food. These efforts have taken the form of subsidies to the airfreight costs of fresh foods, along with educational activities to nurture a greater public awareness of food preparation and healthy eating practices. A government-sponsored Nunavut Harvester’s Support Program assists hunters in obtaining the equipment they need to operate in an environment that is changing from season to season.^19^ Among the proposals, Dewailly observes, has been talk of restricting *trans*-fats in foods. Denmark did so in 2003, which may help explain why Inuit in Greenland (which imports most of its processed foods from Denmark) exhibited dramatically lower *trans*- fatty acid lipid levels than Nunavik Inuit in one study.^20^

Dewailly remains fundamentally optimistic about the ability of traditional foods to continue defining the identity and health of people in the north. In 2009 news photos of the Canadian Governor General Michaelle Jean tucking into seal heart at a ceremonial occasion in a small Arctic community, for example, children can be seen behind her, eyeing the meat as enviously as kids elsewhere would size up a candy store.^21^ These same children could well harbor a similar passion for pop, chips, and chocolate. According to Dewailly, that simply makes them typical of young people everywhere, taking advantage of a global trade in food to sample new flavors wherever they might be found.

In fact, the various surveys suggest consumption of traditional food may increase with age. Dewailly takes that to mean that after trying out whatever the world has to offer, Inuit youth ultimately return to such food as a staple when they start to build a family.

He concedes that environmental influences will continue to compromise the quality of that food, but not to the point of making it worthless. More problematic, Dewailly states, will be mustering the social, political, and economic will to keep providing that food, and to ensure “food sovereignty” among Inuit—the right to shape their own modern diet in a way that is both culturally and physiologically nourishing.

## Figures and Tables

**Figure:**
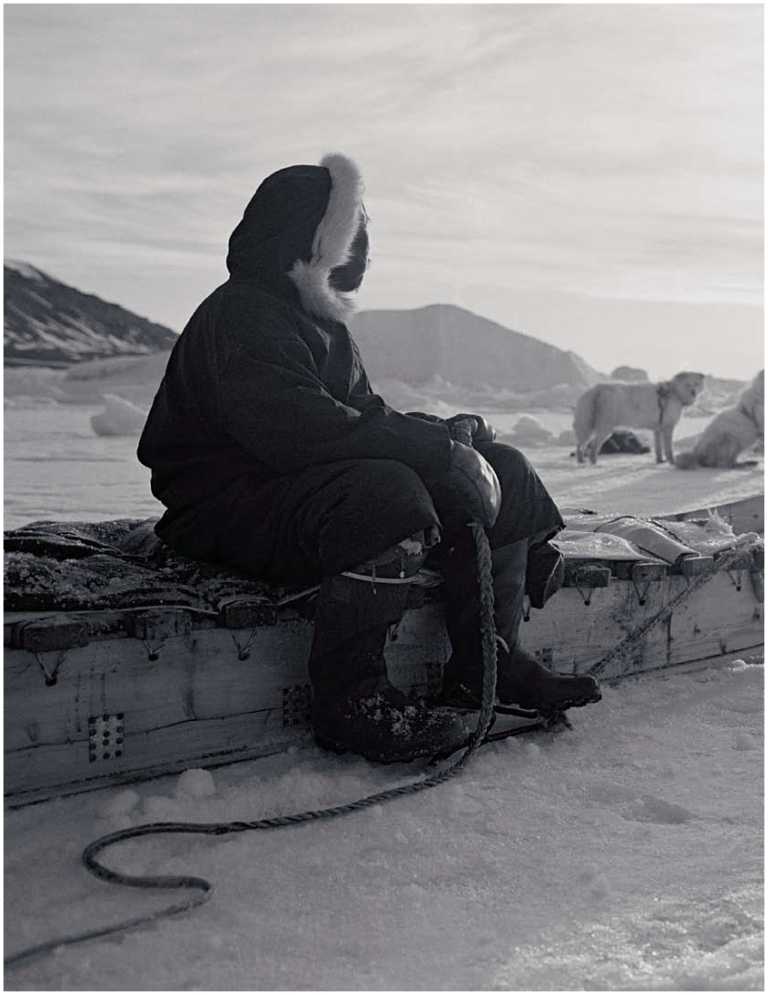


**Figure:**
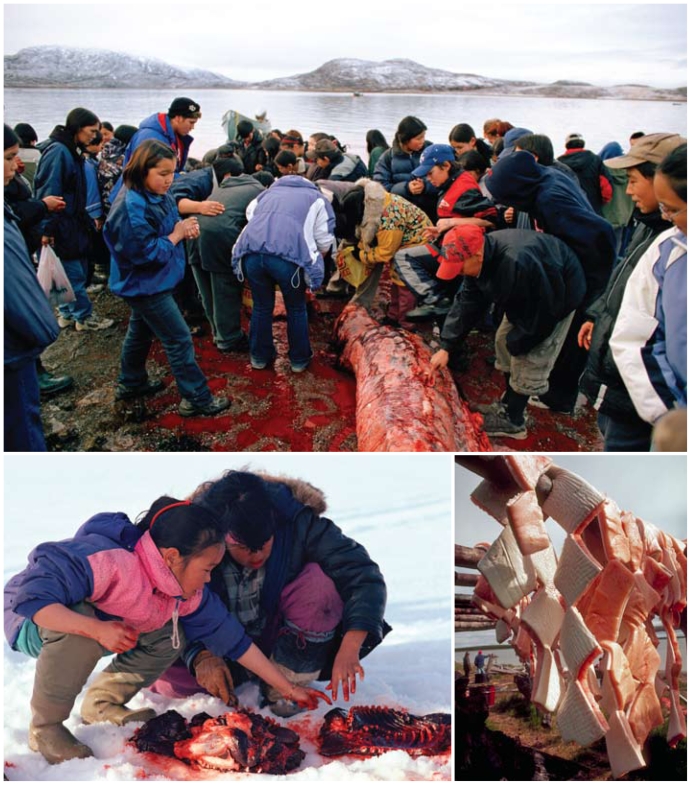
Clockwise from top: Inuit from Cape Dorset gather around the carcass of a beluga whale; carefully cut beluga blubber hangs to dry on wooden racks at an Inuit whaling camp; Inuit girls eat raw seal liver on a summer hunting trip. Opposite: an Inuit hunter prepares for a seal hunt in Cumberland Sound. The forced settlement of Canada’s Inuit following World War II disrupted a nomadic way of life that was perfectly adapted to the Arctic environment. Some communities still observe the mores of “country food,” but an altered economic and sociocultural matrix means many others are losing these traditions. Increased costs to hunt, changing food-sharing relationships, and adherence to a money-based economy (which means employed Inuit have less time to hunt) are some of the barriers communities see to consuming traditional food.^4^

**Figure:**
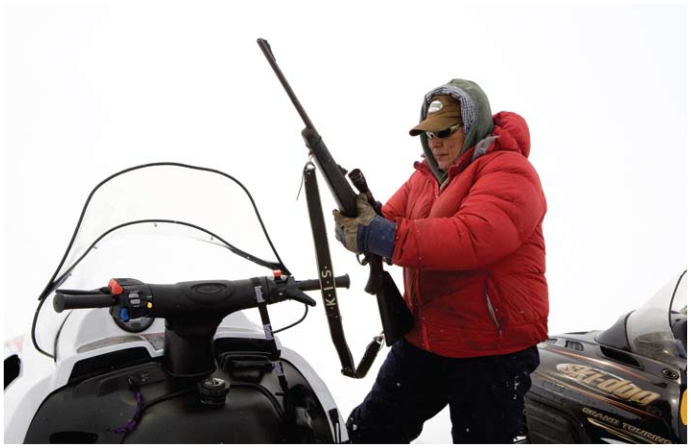


**Figure:**
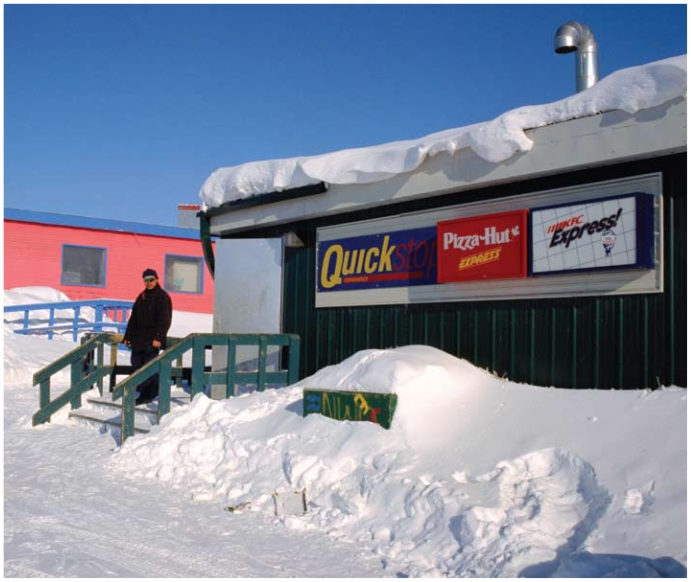
Fast food makes inroads into Iqaluit, the provincial capital of Nunavut. Double-pepperoni pizza and fried chicken provide a far different fat from the animal meat that for millennia met all the nutritional needs of Inuit.

**Figure:**
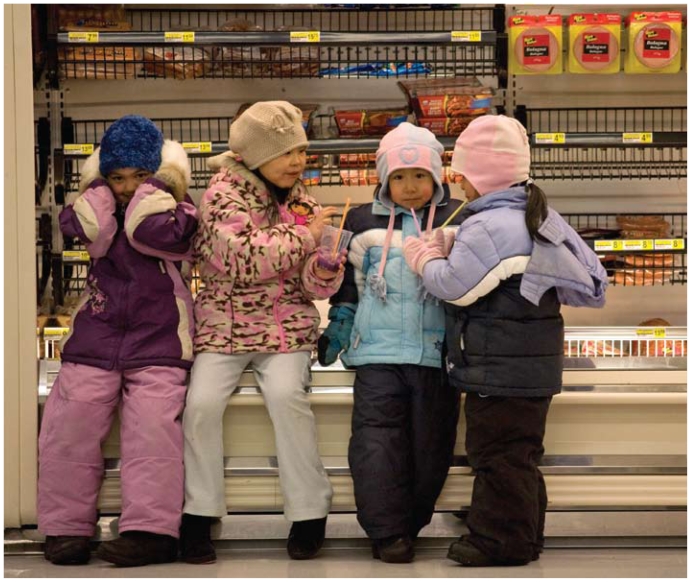
Inuit girls enjoy frozen drinks in a market in Igloolik, Nunavut. In one study of indigenous children 21% of food energy came from store-bought foods identified as “fat” and another 20% from foods identified as “sweet.”^15^

**Figure:**
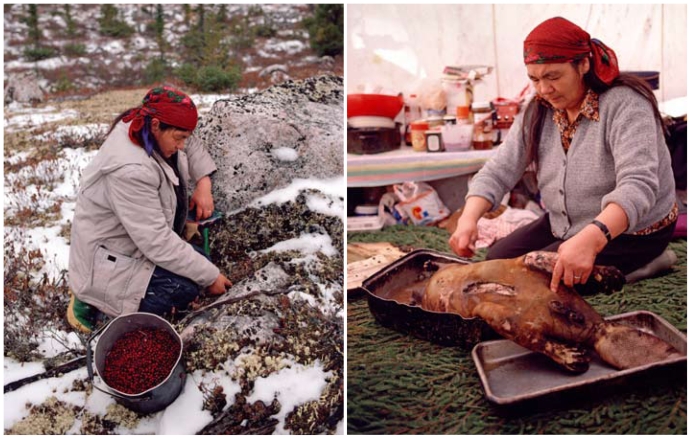
A Cree woman picks cranberries and prepares a beaver for a family feast in northern Quebec, Canada. All is not lost as far as traditional food goes: one survey revealed that two-thirds of indigenous households got at least half their food from traditional sources.^13^
